# Development of Pelvic Abscess Following Water-Skiing Injury

**DOI:** 10.1155/S1064744993000122

**Published:** 1993

**Authors:** Mark D. Pearlman, Lauren Zoschnick

**Affiliations:** 1 University of Michigan Medical Center Ann Arbor MI USA; 2 1500 E. Medical Center Dr., D2202 MPB Box 0718 Ann Arbor MI 48109 USA

## Abstract

Several descriptions of hydrostatic injuries while water-skilng have been described, including lacerations of the perineum, vagina, and cervix. Salpingitis or pelvic abscess resulting from water-skiing injuries are rare but important complications. A case of a pelvic abscess following a fall while water-skiing is described. The abscess was drained laparoscopically, resulting in a good clinical outcome. The mechanism of injury and recommendations for prevention are also presented. Upper genital tract infection may result from water-skiing injuries due to hydrostatic pressure forcing bacteria and water through the vagina and cervix into the endometrium, fallopian tube, and peritoneal cavity. While an uncommon complication, physicians and other practitioners caring for women should be aware of this potential complication from water-skiing.


Infectious Diseases in Obstetrics and Gynecology 1:49-50 (1993)
© 1993 Wiley-Liss, Inc.

### Development of Pelvic Abscess Following Water-Skiing Injury

**Mark D. Pearlman and Lauren Zoschnick**
*University of Michigan Medical Center, Ann Arbor, MI*

ABSTRACT

**Several descriptions of hydrostatic injuries while water-skiing have been described, including lacerations of the perineum, vagina, and cervix. Salpingitis or pelvic abscess resulting from water-skiing injuries are rare but important complications. A case of a pelvic abscess following a fall while water-skiing is described. The abscess was drained laparoscopically, resulting in a good clinical outcome. The mechanism of injury and recommendations for prevention are also presented. Upper genital tract infection may result from water-skiing injuries due to hydrostatic pressure forcing bacteria and water through the vagina and cervix into the endometrium, fallopian tube, and peritoneal cavity. While an uncommon complication, physicians and other practitioners caring for women should be aware of this potential complication from water-skiing.** © 1993 Wiley-Liss, Inc.

KEY WORDS
Wounds and injuries, salpingitis, douche

### CASE REPORT

A 29-year-old woman (G0) was transported to the University of Michigan Emergency Center approximately 2 hours after losing her balance and falling backwards while water skiing. Her initial complaints included nausea, vomiting, and lower back pain. On admission, she was conscious with stable vital signs, and a temperature of 99.1°F. The abdomen was soft with minimal tenderness in both lower quadrants, but no peritoneal signs. No vaginal bleeding or lacerations were noted. Pelvic examination revealed minimal lower abdominal tenderness, but no significant adnexal tenderness or masses were detected. The uterus was normal in size, anteverted, and nontender. Her past medical history was significant for a 3 year history of primary infertility. Laparoscopy performed approximately 2 months before her injury revealed stage II endometriosis and a right ovarian luteoma. The endometriosis was treated with laparoscopic laser ablation, and she recovered uneventfully.

Abdominal radiographs made at the time of admission revealed a nonspecific bowel gas pattern. A computerized tomography (CT) scan of the abdomen and pelvis in the emergency center was normal without evidence of free fluid in the abdomen. She was admitted overnight for observation and discharged the next day in stable condition.

Five days following discharge, the patient was seen by her family practitioner for a febrile illness, and "strep pharyngitis" was diagnosed. She was treated with penicillin V tablets, 250 mg for 7 days. Three days following initiation of this treatment, she returned to her family practitioner with persistent fever, and increasing left lower abdominal pain. Pelvic inflammatory disease was diagnosed and she was started on oral metronidazole and doxycycline. Two days later she was seen at the University of Michigan Hospital with the complaint of increasingly severe bilateral lower abdominal pain. She denied being sexually active since, or for 2 weeks prior to the injury. On this admission her vital signs were stable with a temperature of

Address correspondence/reprint requests to Dr. Mark D. Pearlman, 1500 E. Medical Center Dr., D2202 MPB; Box 0718, Ann Arbor, MI 48109.

Received December 8, 1992
Accepted February 26, 1993

99.9°F. Tenderness and guarding in both lower quadrants with rebound tenderness were found on physical examination. Bimanual examination of the pelvis revealed a tender 9 cm left adnexal mass. The admitting WBC count was 14,900/$mm^3$ with a hematocrit of 36.1%, a Westegren sedimentation rate of 58 mm/sec, and a normal urinalysis. Chlamydia and gonorrhea cultures of the cervix were negative. A pelvic ultrasound revealed a 9 × 6 × 6 cm complex cystic mass in the left adnexa. She was admitted with a presumed left pelvic abscess and was begun on parenteral ampicillin, gentamicin, and metronidazole. The following day, a laparoscopic examination revealed a left pelvic abscess involving the ovary, left fallopian tube, bowel, and omentum. The abscess was drained and the pelvis was thoroughly irrigated through the laparoscope. A closed drainage system was placed in the region and brought out through the abdominal wall. Parenteral antibiotic therapy was continued for a total of 8 days, and was discontinued after the patient had been afebrile for 48 hours. The WBC count on discharge was 10,300/$mm^3$. Oral amoxicillin/clavulinic acid, 500 mg TID, was prescribed for 10 days. Aerobic and anaerobic cultures of the purulent fluid at laparoscopy revealed only a moderate quantity of Group C streptococci. She has done well since discharge.

### DISCUSSION

Hydrostatic injury of the perineum, vagina, vulva, bladder, rectum, and descending colon have been described.[1–4] Most vaginal injuries resulting from water-skiing accidents involve the mechanism of falling backwards and striking the perineum on the water, which may force water through the vagina and cervix into the upper genital tract or peritoneal cavity. While a few prior descriptions of salpingitis following water-skiing injury have been presented, we can find no report of a resultant pelvic abscess.[5–7]

The hydrostatic pressure of this water likely forces bacteria present both in the water and in the vagina and endocervix into the upper genital tract. This douche of lake water under high pressure probably traversed the lower and upper genital tract into the peritoneal cavity, causing the immediate onset of symptoms. The initial event was followed by multiplication of bacteria in the region of inoculation, eventually resulting in pelvic abscess. The actual microbiology of this abscess was probably altered by the antibiotics administered before laparoscopy, as it is reasonable to assume that this was initially a polymicrobial infection.

It is unlikely that this was a preexisting abscess or that it developed from a sexually transmitted infection following the injury, as there was documentation of a normal left adnexa on laparoscopy 2 months prior, a normal pelvic CT scan on the day of the injury, and self-described abstinence since the injury.

While not common, douche-induced water-skiing injuries of the perineum, including laceration of the lower genital tract, rectum, and descending colon, have been described. These accidents may also result in infections of the upper genital tract or pelvis. The risk of injury could be substantially reduced by wearing rubber wet suits to prevent direct hydrostatic injury. It would seem prudent to recommend the use of protective garments of this type for all water-skiers. Manufacturers of water skis could provide this information in an insert provided with the purchase of all new skis, or a small sticker on each ski suggesting the use of a rubber wet suit while skiing, particularly in women. Gynecologists, emergency medicine physicians, surgeons, and other providers of health care for women should be aware of potential water pressure injuries following water-skiing.

### REFERENCES

1. Niv J, Lessing JB, Hartuv J, Peyser MR: Vaginal injury from sliding down a water chute. Am J Obstet Gynecol 166:930–931, 1992.
2. Tweedle PG: Gynecologic hazards of water-skiing. CMA 108:20–21, 1973.
3. Edington RF: Vaginal injuries due to water-skiing. CMA 119:310–311, 1978.
4. Ramey JR: Intrarectal tear with bleeding from water-skiing accident. J Fla Med Assoc 61:162, 1974.
5. Pfanner D: Salpingitis and water-skiing. Med J Aust I:320, 1964.
6. Morton DC: Gynaecological complications of water-skiing. Med J Aust II:1256–1257, 1970.
7. Brisbane gynaecologist: Dangers of water-skiing. Med J Aust I:646, 1968.
